# Hölder norm estimate for the fractal Hilbert transform in Douglis analysis

**DOI:** 10.1186/s13660-017-1488-7

**Published:** 2017-09-08

**Authors:** Yudier Peña Pérez, Martín P Árciga Alejandre, Ricardo Abreu Blaya, Juan Bory Reyes

**Affiliations:** 1 Facultad de Matemática, Universidad Autónoma de Guerrero, Avenida Lázaro Cárdenas sn, Chilpancingo, Guerrero 39087 México; 2 Departamento de Matemática, Universidad de Holguín, Avenida XX Aniversario, Holguín, 80100 Cuba; 3 SEPI-ESIME-ZAC, Instituto Politécnico Nacional, Col. Linda Vista, Delegación Gustavo A. Madero, Ciudad México, 07738 México

**Keywords:** hyperanalytic functions, fractal dimensions, Whitney decomposition, Hilbert transform, Hölder functions, *h*-summables curves

## Abstract

The main goal of this paper is to estimate the Hölder norm of a fractal version of the Hilbert transform in the Douglis analysis context acting from Hölder spaces of Douglis algebra valued functions defined on *h*-summable curves.

## Introduction

Douglis analysis is an alternative approach to complex methods for the investigation of linear and uniformly elliptic systems of 2*n* equations for 2*n* desired real-valued functions.

The function theory associated with the Douglis operator in $\mathbb {R}^{2}$ (identifying $\mathbb{R}^{2}$ with $\mathbb{C}$ in the usual way) plays a very important role in problems in pure mathematics, mathematical physics, and engineering, such as plane elasticity theory and hydromechanics.

The well-known Douglis system, that is, an elliptic system of first order in two independent variables, can be represented by a single “hypercomplex” equation. Solutions of such equation (null solutions of the Douglis system) are termed hyperanalytic functions. In [1] Douglis presented a complete study of the hyperanalytic function theory. For greater details the reader is directed to [2] and to [3] for a thorough treatment of this theory.

In more recent times hyperanalytic function theory has been developed for solving problems of mathematical physics such as plate and shell problems. In [4, 5] the authors provided conditions for the solvability of the Riemann boundary value problem for hyperanalytic functions on classes of fractal closed curves. Hence, this can be regarded of as a good motivation for finding conditions on the boundary, which give boundedness of certain singular integral operators, such as the Hilbert transform when the boundary is permitted to be fractal. To this end, in [6] the authors gave an estimate for the upper bound of the Hölder norm a fractal version of the Hilbert transform for domains with *d*-summable boundary; a geometric notion introduced in [7], which is essential for integration of a form over a fractal boundary.

Serving as a generalization of the concept of *d*-summability, the authors of [5] proposed a novel modification by the use of a gauge function (dimension function) in order to use different functions of diameter. Explicit examples were given to illustrate how the notion of *h*-summability can be applied to describe the geometry of a simply connected bounded open subset of the plane in a more delicate manner than the latter one. Several geometric facts related to *d*-summable sets can be generalized to *h*-summable sets.

The present paper aims to give an explicit expression for the upper bound of the norm of a fractal version of the Hilbert transform involving domains with *h*-summable boundary. This makes our results much more general to that given in [6].

## Preliminaries

In this section we set up notation and terminology on Douglis analysis and fractal geometry to be used throughout the paper.

### Douglis algebras and hyperanalytic functions

We consider the Douglis algebra $\mathbb{D}$ generated by *i* and *e*, subjected to the multiplication rules

$$ i^{2}=-1,\qquad e^{0}=1,\qquad ie=ei,\qquad e^{r}=0, $$

where *r* is a positive integer.

Any arbitrary element $a\in\mathbb{D}$ is a hypercomplex number of the form

$$ a=\sum_{k=0}^{r-1}a_{k}e^{k}, $$

where each $a_{k}$ is a complex number, $a_{0}$ is called the complex part of *a* and $A=\sum_{k=1}^{r-1}a_{k}e^{k}$ the nilpotent part.

It is possible to introduce the conjugate element of *a* which is defined as

$$ \bar{a}:=\sum_{k=0}^{r-1}\bar{a_{k}}e^{k}, $$

and the norm of *a* is defined by $\Vert a \Vert :=\sum_{k=0}^{r-1} \vert a_{k} \vert $. Note that the Douglis algebra is commutative and associative.

The multiplicative inverse $a^{-1}$ of *a* with complex part $a_{0}\neq 0$ is given by

$$ a^{-1}=a_{0}^{-1}\sum_{k=0}^{r-1}(-1)^{k} \biggl( \frac {A}{a_{0}} \biggr) ^{k}. $$

Observe that if $a_{0}=0$, then *a* does not have multiplicative inverse and is called nilpotent. The Douglis analysis is then the study of the Douglis algebra valued functions. Let *f* be a $\mathbb{D}$-valued function (hypercomplex function) then *f* may be written as $f=\sum_{k=0}^{r-1}f_{k}e^{k}$, where $f_{k}$ are complex-valued functions.

The Douglis operator $\partial_{\bar{z}}^{J}$ is given by

$$ \partial_{\bar{z}}^{J}:=\partial_{\bar {z}}+J(z) \partial_{z},\quad z=x+iy, $$

where $J(z)$ is a known nilpotent hypercomplex function and

$$ \partial_{\bar{z}}:=\frac{1}{2}(\partial _{x}+i \partial_{y}),\qquad \partial_{z}:=\frac{1}{2}( \partial_{x}-i\partial_{y}). $$

Suppose $\Omega\in\mathbb{C}$ to be a domain, a smooth hypercomplex function *f* defined in Ω is said to be hyperanalytic in Ω if $\partial_{\bar{z}}^{J}f=0$ in Ω. As an example for hyperanalytic function we take the generating solution of the Douglis operator, see [2, Section 2, p.11], given by

$$ W(z)=z+\sum_{k=1}^{r-1}W_{k}(z)e^{k}, $$

where its nilpotent part posses bounded and continuous derivate up to order two in $\mathbb{C}$. Other important example for hyperanalytic function is the so-called hypercomplex Cauchy kernel, *i.e.*, the fundamental solution of the Douglis operator, given by

$$ e_{z}(\zeta):=\frac{1}{2\pi}\frac{\partial_{\zeta }W(\zeta)}{W(\zeta)-W(z)},\quad\zeta\neq z. $$

The singularity of $e_{z}(\zeta)$ at $\zeta=z$ is the same as $\frac {1}{\zeta-z}$.

We continue this section by compiling some of the important facts of fractal geometry.

### Summable sets in $\mathbb{C}$

By definition, presented in [7], a set $\mathbf{E}\subset\mathbb {R}^{2}$ is said to be *d*-summable if the improper integral

$$ \int_{0}^{1}N_{\mathbf{E}}(t)t^{d-1}\,dt, $$

converges, where $N_{\mathbf{E}}(t)$ stands for the least number of *t*-balls needed to cover **E**.

A quite natural generalization, inspired by the idea of a gauge function (dimension function), may be obtained by the use of different functions of diameter than just $t^{d}$ and requiring the convergence of the corresponding improper integral.

Let **E** be an arbitrary bounded subset of $\mathbb{R}^{2}$, whose diameter will be denoted by $\vert {\mathbf{E}} \vert $.

#### Definition 1

[8, Definition 1]

Let *h* be a finite, positive, non-decreasing function in $(0,+\infty )$ with $\lim_{t\to0+}h(t)=0$. The set **E** is said to be *h*-summable if the improper integral

$$ \int _{0}^{\delta}N_{\mathbf{E}}(t)\frac{h(t)}{t} \,dt $$

converges, for some $\delta>0$.

The notion of *h*-summable set remains unchanged if $N_{\mathbf{E}}(t)$ is taking as the number of *k*-squares needed to cover **E** with $2^{-k}\leq t<2^{-k+1}$. A square *Q* is called a *k*-square if it is of the form

$$[l_{1}2^{-k},(l_{1}+1)2^{-k}] \times \bigl[l_{2}2^{-k},(l_{2}+1)2^{-k} \bigr], $$

where *k*, $l_{1}$, $l_{2}$ are integers.

Of course, **E** is *d*-summable if it is *h*-summable with $h(t)=t^{d}$. Several geometric facts related to *d*-summable sets can be generalized to *h*-summable sets; see [8].

In what follows, let $\Omega\subset\mathbb{C}$ be a Jordan domain, the boundary of which is denoted by *γ*. The following lemma is a generalization of that appeared in [7] and reveals the specific importance of the *h*-summability.

#### Lemma 1

[8, Lemma 1]

*If* Ω *is a Jordan domain of* $\mathbb{C}$ *and its boundary* *γ* *is* *h*-*summable*, *then the expression* $s(h):=\sum_{Q\in \mathcal{W}}h( \vert Q \vert )$, *called* *h*-*sum of the Whitney decomposition* $\mathcal{W}$ *of* Ω, *is finite*.

For further details of the Whitney decomposition we refer to [9]. We write $s(d)$ instead of $s(h)=\sum_{Q\in\mathcal{W}} \vert Q \vert ^{d}$, when $h(t)=t^{d}$. If *γ* is a rectifiable curve, the following useful lemma holds.

#### Lemma 2

[6, Lemma 1.2]

*If* *γ* *is a rectifiable curve with length* $\mathbf{l}[\gamma]$, *then for every* $\varepsilon>0$

1 $$ s(1+\varepsilon)\le c\frac{\mathbf{l}[\gamma]}{\varepsilon}. $$

Here and subsequently, *c* will denote a positive constant, not necessarily the same at different occurrences.

### Functional spaces in $\mathbb{C}$

A positive real function *ϕ* defined in $(0,+\infty)$, with $\lim_{t\to0+}\phi(t)=0$ is said to be a majorant if $\phi(t)$ is non-decreasing and $\frac{\phi(t)}{t}$ is non-increasing.

If in addition

$$ \int^{\delta}_{0}\frac{\phi(t)}{t} \,dt + \delta \int^{\infty}_{\delta}\frac{\phi(t)}{t^{2}} \,dt \leq c \phi ( \delta), $$

for a suitable constant $c=c(\phi)$ and $\delta\in(0,1)$, then we will say that *ϕ* belongs to the Bari-Stechkin class Φ.

Let us recall that a non-negative function *ϕ* is said to be almost increasing (or almost decreasing) if there exists a constant $c\geq1$ such that $\phi(x)\le c\phi(y)$ for all $x\le y$ ($y\le x$, respectively). Note that functions of the form $\phi(t)=t^{\alpha}$, $0<\alpha<1$, belong to Φ.

#### Remark 1

According to [10, Lemma 3.3] we can ensure that $\phi\in\Phi $ if and only if there exist numbers $\alpha_{\phi}$, $\beta_{\phi }$ in $(0,1)$ such that the functions $\frac{\phi(t)}{t^{\alpha _{\phi}}}$ and $\frac{\phi(t)}{t^{\beta_{\phi}}}$ are almost decreasing and almost increasing in $(0,+\infty]$, respectively.

Therefore, a constant $c>0$ may be found to guarantee the relation

$$ c^{-1}t^{\alpha_{\phi}}\leq\phi(t)\leq c t^{\beta_{\phi}}. $$

We will denote by $\mathcal{H}_{\varphi}(\mathbf{E})$ the set of all generalized Hölder continuous hypercomplex functions *f* for which

$$ \vert f \vert _{\varphi,\mathbf{E}}:=\sup_{x,y\in{\mathbf{E}}, x\neq y} \frac{ \Vert f(x)-f(y) \Vert }{\varphi( \vert x-y \vert )}< \infty, $$

where $\varphi\in\Phi$. For example, $\varphi(t)=t^{\nu}$, $t\in (0, \vert \mathbf{E} \vert ]$, $0<\nu\leq1$, is a majorant and we have the usual Hölder class $\mathcal{H}_{\nu}(\mathbf{E})$.

One can define

$$ \Vert f \Vert _{\varphi,\mathbf{E}}:= \vert f \vert _{\varphi,\mathbf{E}}+\max _{x\in\mathbf{E}} \bigl\Vert f(x) \bigr\Vert . $$

In closing this introductory section let us remember that a Whitney extension (see [9]) of $f\in\mathcal{H}_{\varphi}(\mathbf {E})$, $\mathbf{E}\subset\mathbb{C}$ being compact, is a function $\mathcal{E}_{0}(f)$ that belongs to $\mathcal{H}_{\varphi}(\mathbb {C})$ and has partial derivatives of all orders at any point $z \in \mathbb{C}\setminus\mathbf{E}$. Moreover, there exists a constant $c>0$ such that

2 $$ \bigl\Vert \partial_{\bar{z}}^{J} \mathcal{E}_{0}(f) (z) \bigr\Vert \leq c \vert f \vert _{\varphi,\mathbf{E}}\frac{\varphi (\operatorname {dist}(z,\mathbf{E}))}{\operatorname {dist}(z,\mathbf{E})},\quad z \in\mathbb {C}\setminus{\mathbf{E}}. $$

Here and in the sequel we denote by $\operatorname {dist}(A,B)$ the distance between sets *A* and *B*.

If ${\mathcal{X}}(z)$ denotes the characteristic function of the set $\Omega\cup\gamma$, we shall write $f^{\omega}(z):={\mathcal {X}}(z)\mathcal{E}_{0}(f)(z)$.

## The hypercomplex Cauchy type integral on *h*-summable curves

In this section we define and characterize the hypercomplex Cauchy type integral on *h*-summable curves. This definition is inspired by the Borel-Pompeiu formula derived in [2, Theorem 1.3]. We deal with an appropriate extension for hypercomplex functions *f* defined on a *h*-summable curve *γ*, which is obtained by the Whitney extension operator $\mathcal{E}_{0}$.

### Definition 2

Let $\varphi\in\Phi$ and Ω be a domain with *h*-summable boundary *γ* where $h(t)=\varphi(t)t$, for $t \in[0, \vert \gamma \vert ]$. We define the Cauchy type integral of $f\in\mathcal{H}_{\varphi}(\gamma)$ by the formula

3 $$ \bigl(\mathbf{C}^{*}_{\gamma}f\bigr) (z)=f^{\omega}(z)-T_{\Omega}\bigl[\partial _{\bar{z}}^{J} \mathcal{E}_{0}f\bigr](z), \quad z\in\mathbb{C}\setminus\gamma, $$

where

$$ T_{\Omega}\bigl[\partial_{\bar{z}}^{J}\mathcal{E}_{0}f \bigr](z):= \int_{\Omega }e_{z}(\zeta)\partial_{\bar{\zeta}}^{J} \mathcal{E}_{0}(f) (\zeta)\, d\xi\,d\eta,\quad \zeta=\xi+i\eta. $$

The following proposition makes this definition legitimate.

### Proposition 1

*The hypercomplex function* (3) *is correctly defined for any* $z\in\mathbb{C}\setminus\gamma$ *and its value does not depend on the particular choice of* $\mathcal{E}_{0}(f)$

### Proof

It is enough to prove that

$$ \int_{\Omega} \bigl\Vert \partial_{\overline{\zeta }}^{J} \mathcal{E}_{0}(f) (\zeta) \bigr\Vert \,d\xi\,d\eta< \infty. $$

We follow [9] considering the Whitney decomposition of Ω, $\mathcal{W}=\bigcup_{k}\mathcal{W}^{k}$, which consists of disjoint squares *Q* satisfying

$$ \vert Q \vert \le \operatorname {dist}(Q,\gamma)\le4 \vert Q \vert . $$

Then we have

$$ \begin{aligned} \int_{\Omega} \bigl\Vert \partial_{\overline{\zeta }}^{J} \mathcal{E}_{0}(f) (\zeta) \bigr\Vert \,d\xi\,d\eta&=\sum _{Q\in\mathcal{W}} \int_{Q} \bigl\Vert \partial_{\overline{\zeta }}^{J} \mathcal{E}_{0}(f) (\zeta) \bigr\Vert \,d\xi\,d\eta \\ &\leq c\sum_{Q\in\mathcal{W}} \int_{Q} \bigl(\operatorname {dist}(\zeta,\gamma)\bigr)^{-1}\varphi \bigl(\operatorname {dist}(\zeta,\gamma)\bigr)\,d\xi\,d\eta, \end{aligned} $$

where the last inequality is a consequence of (2). Taking account that $\frac{\varphi(t)}{t}$ does not increase, we have

4 $$ \frac{\varphi(\operatorname {dist}(\zeta,\gamma))}{\operatorname {dist}(\zeta,\gamma)}\leq\frac {\varphi(\operatorname {dist}(Q,\gamma))}{\operatorname {dist}(Q,\gamma)}\leq\frac{\varphi( \vert Q \vert )}{ \vert Q \vert } $$

for all $\zeta\in Q$.

Consequently

$$ \int_{\Omega} \bigl\Vert \partial_{\overline{\zeta }}^{J} \mathcal{E}_{0}(f) (\zeta) \bigr\Vert \,d\xi\,d\eta\leq c\sum _{Q\in\mathcal{W}} \varphi\bigl( \vert Q \vert \bigr) \vert Q \vert =c\sum_{Q\in \mathcal{W}}h\bigl( \vert Q \vert \bigr)\leq c. $$

At this stage we are reduced to Lemma 1, concerning the finiteness of the sum $\sum_{Q\in\mathcal{W}}h( \vert Q \vert )$.

Now suppose that $\mathcal{E}^{1}_{0}(f)$ and $\mathcal{E}^{2}_{0}(f)$ are two different Whitney extensions of *f*. Then $\mathcal {E}_{0}(g):=\mathcal{E}^{1}_{0}(f)-\mathcal{E}^{2}_{0}(f)$, is a Whitney extension of the null function and hence $\mathcal{E}_{0}(g)| _{\gamma}=0$. If we prove that the hypercomplex function

5 $$ g^{\omega}(z)- \int_{\Omega}e_{z}(\zeta)\partial_{\bar{\zeta }}^{J} \mathcal{E}_{0}(g) (\zeta)\,d\xi\,d\eta $$

vanishes in $\mathbb{C}\setminus\gamma$, the assertion follows.

Define

$$ \Omega_{k}=\bigl\{ \underline{x}\in Q: Q\in\mathcal{W}^{j} \mbox{ for some } j\le k\bigr\} $$

and $\Delta_{k}=\Omega\setminus\Omega_{k}$. The boundary of $\Omega _{k}$, denoted by $\gamma_{k}$, is composed of certain sides of some squares $Q\in\mathcal{W}^{k}$. We have

6 $$ \int_{\Omega}e_{z}(\zeta)\partial_{\bar{\zeta}}^{J} \mathcal {E}_{0}(g) (\zeta)\,d\xi\,d\eta= \lim_{k\rightarrow\infty} \biggl( \int _{\Omega_{k}} + \int _{\Delta _{k}}\biggr)e_{z}(\zeta)\partial_{\bar{\zeta}}^{J} \mathcal{E}_{0}(g) (\zeta )\,d\xi\,d\eta. $$

Let $z\in\Omega$ and let $k_{0}$ be so large chosen such that $z\in \Omega_{k_{0}}$ and $\operatorname {dist}(z,\gamma_{k})\ge \vert Q_{0} \vert $ for $k>k_{0}$, where $Q_{0}$ is a square of $\mathcal {W}^{k_{0}}$. By the Borel-Pompeiu formula we deduce

7 $$ g^{\omega}(z)- \int _{\Omega_{k}}e_{z}(\zeta)\partial_{\bar{\zeta }}^{J} \mathcal{E}_{0}(g) (\zeta)\,d\xi\,d\eta= \int _{\gamma_{k}} e_{z}(\zeta)n_{J}( \zeta)g^{\omega}(\zeta)\,ds , \quad z\in\Omega_{k}, $$

where $n_{J}(\zeta)=n(\zeta)+{\overline{n}}(\zeta)J(\zeta)$ being $n(\zeta)$ the exterior unit normal vector at the point *ζ* on *γ* in Federer’s sense (see [11]), and *ds* denotes the arclength differential.

Next, let $\zeta\in\gamma_{k}$, $Q\in{\mathcal{W}}^{k}$ a square containing *ζ*, and $\zeta_{*}\in\gamma$ such that $\vert \zeta-\zeta_{*} \vert =\operatorname {dist}(\zeta,\gamma)$.

Since $\mathcal{E}_{0}(g)| _{\gamma}=0$, we have

$$ \bigl\Vert \mathcal{E}_{0}(g) (\zeta) \bigr\Vert = \bigl\Vert \mathcal {E}_{0}(g) (\zeta)-\mathcal{E}_{0}(g) ( \zeta_{*}) \bigr\Vert \le c\varphi \bigl( \vert \zeta- \zeta_{*} \vert \bigr) \le c \varphi \bigl( \vert Q \vert \bigr) . $$

If Σ denotes a side of $\gamma_{k}$ and $Q\in\mathcal{W}^{k}$ is the *k*-square containing Σ, we have for $k>k_{0}$

$$ \biggl\Vert \int _{\Sigma} e_{z}(\zeta)n_{J}(\zeta )g^{\omega}(\zeta)\,ds \biggr\Vert \le\frac{c}{ \vert Q_{0} \vert } \int _{\Sigma} \bigl\Vert g^{\omega}(\zeta) \bigr\Vert \, ds\le\frac{c}{ \vert Q_{0} \vert }\frac{\varphi( \vert Q \vert )}{ \vert Q \vert ^{-1}}. $$

As noticed before, each side of $\gamma_{k}$ is one of those 4 of some $Q\in\mathcal{W}^{k}$. Therefore, for $k>k_{0}$

$$ \biggl\Vert \int _{\gamma_{k}} e_{z}(\zeta )n_{J}( \zeta)g^{\omega}(\zeta)\,ds \biggr\Vert \le\frac{c}{ \vert Q_{0} \vert }\sum _{Q\in\mathcal{W}^{k}}{\varphi\bigl( \vert Q \vert \bigr)} { \vert Q \vert }. $$

The finiteness of $\sum_{Q\in\mathcal{W}}h( \vert Q \vert )$ [8, Lemma 1] implies that

$$ \lim_{k\rightarrow\infty} \int _{\gamma_{k}} e_{z}(\zeta)n_{J}( \zeta)g^{\omega}(\zeta)\,ds=0. $$

Combining (6) with (7) shows that (5) vanishes for $z\in\Omega$.

The same conclusion is obtained for $z\in\mathbb{C}\setminus\{{\Omega \cup\gamma}\}$. The key idea is to note that $\operatorname {dist}(z,\gamma _{k})\ge \operatorname {dist}(z,\gamma)$ for $z\in\mathbb{C}\setminus\{ {\Omega\cup\gamma}\}$. □

A natural question to ask is whether $\mathbf{C}^{*}_{\gamma}f$ has a continuous extension to $\Omega\cup\gamma$. It is generally a highly nontrivial question. However, on the positive side, the next theorem sheds some light on the answer and one can therefore also introduce the following fractal hypercomplex Hilbert transform:

$$ \bigl(H^{*}_{\gamma}f\bigr) (t):=2\bigl({\mathbf{C}^{*}_{\gamma }}^{+}f \bigr) (t)-f(t),\quad t\in\gamma. $$

Here ${\mathbf{C}^{*}_{\gamma}}^{+}f$ denotes the trace on *γ* of the continuous extension of $\mathbf{C}^{*}_{\gamma}f$ to $\Omega \cup\gamma$. This approach is an alternative to the more conventional hypercomplex Hilbert transform, which is defined to be the Cauchy principal value singular integral

$$ (H_{\gamma}f) (t):= \int_{\gamma}e_{t}(\zeta ) \bigl(f(\zeta)-f(t)\bigr)\,d \zeta+f(t),\quad t\in\gamma. $$

### Theorem 1

*Let* *φ* *and* *ψ* *be given in* Φ *with* $\alpha_{\psi}\le \beta_{\varphi}$ (*see Remark* 1) *and let* *γ* *be an* *h*-*summable curve with* $h(t)=\varphi(t)^{p}t^{2-p}$ *for* $p= \frac {2}{1-\alpha_{\psi}}$. *Then for any function* $f\in\mathcal {H}_{\varphi}(\gamma)$ *the Cauchy type integral* ${\mathbf {C}^{*}_{\gamma}}f$ *has a continuous extension to* ${\Omega\cup\gamma }$. *Furthermore*, $H^{*}_{\gamma}f\in\mathcal{H}_{\psi}(\gamma)$.

### Proof

We have

$$ \begin{aligned} \int_{\Omega} \bigl\Vert \partial_{\bar{\zeta}}^{J} \mathcal {E}_{0}(f) (\zeta) \bigr\Vert ^{p} \,d\xi\,d\eta&= \sum_{Q\in\mathcal {W}} \int_{Q} \bigl\Vert \partial_{\bar{\zeta}}^{J} \mathcal {E}_{0}(f) (\zeta) \bigr\Vert ^{p} \,d\xi\,d\eta \\ &\leq c \vert f \vert _{\varphi,\gamma}^{p} \sum _{Q\in \mathcal{W}} \int_{Q}\bigl(\operatorname {dist}(\zeta,\gamma)^{-1}\varphi \bigl(\operatorname {dist}(\zeta ,\gamma)\bigr)\bigr)^{p}\,d\xi\,d\eta \end{aligned} $$

by the Whitney extension theorem.

Taking into account that $\frac{\varphi(t)}{t}$ does not increase, then

$$\begin{aligned} \bigl(\operatorname {dist}(\zeta,\gamma)^{-1}\varphi\bigl(\operatorname {dist}(\zeta,\gamma)\bigr) \bigr)^{p}\leq\frac {\varphi( \vert Q \vert )^{p}}{ \vert Q \vert ^{p}} \end{aligned}$$

for all $\zeta\in Q$.

Consequently

$$ \begin{aligned} \int_{\Omega} \bigl\Vert \partial_{\bar{\zeta}}^{J} \mathcal {E}_{0}(f) (\zeta) \bigr\Vert ^{p} \,d\xi\,d\eta & \leq c \vert f \vert _{\varphi,\gamma}^{p} \sum _{Q\in\mathcal{W}}\frac {\varphi( \vert Q \vert )^{p}}{ \vert Q \vert ^{p}} \vert Q \vert ^{2}= c \vert f \vert _{\varphi ,\gamma}^{p} \sum _{Q\in\mathcal{W}}\varphi\bigl( \vert Q \vert \bigr)^{p} \vert Q \vert ^{2-p} \\ &= c \vert f \vert _{\varphi,\gamma}^{p} \sum _{Q\in\mathcal {W}}h\bigl( \vert Q \vert \bigr); \end{aligned} $$

the last sum above is finite, which is obtained from the finiteness of the *h*-sum of the Whitney partition.

Since *p* was so chosen to satisfy $p= \frac{2}{1-\alpha_{\psi}}>2$, then it follows from [2, Theorem 1.25] that $T_{\Omega}[\partial _{\bar{z}}^{J}\mathcal{E}_{0}f]$ represents a continuous function in $\mathbb{C}$, which belongs to $\mathcal{H}_{(p-2)/p}(\mathbb {C})=\mathcal{H}_{\alpha_{\psi}}(\mathbb{C})$. This forces $\mathbf {C}^{*}_{\gamma}f$ to admit a continuous extension to $\Omega\cup \gamma$.

On the other hand $f\in\mathcal{H}_{\psi}(\gamma)$, which easily follows from the condition $\alpha_{\psi}\le\beta_{\varphi}$.

The interior limit value of ${\mathbf{C}^{*}_{\gamma}}f$ is then given by

$$ \bigl({\mathbf{C}^{*}_{\gamma}}^{+}f\bigr) (t)=f(t)- T_{\Omega }\bigl[\partial_{\bar{z}}^{J}\mathcal{E}_{0}f \bigr](t), \quad t\in\gamma, $$

whence $H^{*}_{\gamma}f$ is well defined.

The above facts together with Remark 1 finally yield $H^{*}_{\gamma}f\in\mathcal{H}_{\psi}(\gamma)$ and the proof is complete. □

### Hölder norm estimate for $H^{*}_{\gamma}$

In this subsection we show how, under conditions of Theorem 1, the fractal Hilbert transform $H^{*}_{\gamma}$ behaves as a bounded operator acting between the spaces $\mathcal {H}_{\varphi}(\gamma)$ and $\mathcal{H}_{\psi}(\gamma)$. We also estimate its Hölder norm. The main result is of comparable strength to that of [6, Theorem 2.5] for the case of *d*-summables curves.

#### Theorem 2

*If* *φ*, *ψ* *and* *γ* *are as in Theorem* 1, *then* $H^{*}_{\gamma}$ *is bounded from* $\mathcal {H}_{\varphi}(\gamma)$ *into* $\mathcal {H}_{\psi}(\gamma)$ *and*

8 $$ \bigl\Vert H^{*}_{\gamma} \bigr\Vert \le1+ c_{1}\frac{\varphi( \vert \gamma \vert )}{\psi( \vert \gamma \vert )}+c_{2}\bigl(s(h)\bigr)^{\frac{1-\alpha_{\psi}}{2}} \psi\bigl( \vert \gamma \vert \bigr)+c_{3}\bigl(s(h) \bigr)^{\frac{1-\alpha_{\psi}}{2}}, $$

*where* $c_{1}$, $c_{2}$, $c_{3}$ *depend only on* *φ* *and ψ*.

#### Proof

First of all write

$$ \frac{\varphi(t)}{\psi(t)}= \biggl( \frac{\varphi (t)}{t^{\beta_{\varphi}}} \biggr) \biggl( \frac{t^{\beta_{\varphi }}}{t^{\alpha_{\psi}}} \biggr) \biggl( \frac{t^{\alpha_{\psi}}}{\psi (t)} \biggr) . $$

Taking into account the Remark 1 and the above expression, it easily follows that $\frac{\varphi}{\psi}$ is an almost increasing function. This implies that, for $f\in\mathcal {H}_{\varphi}(\gamma)$, we have

$$ \vert f \vert _{\psi,\gamma}\leq c_{1} \frac{\varphi( \vert \gamma \vert )}{\psi( \vert \gamma \vert )} \vert f \vert _{\varphi,\gamma}, $$

where $c_{1}$ depends only on *φ* and *ψ*.

On the other hand, a more careful look at the proof of Theorem 1 reveals that

$$ \begin{aligned} \int_{\Omega} \bigl\Vert \partial_{\bar{\zeta}}^{J} \mathcal {E}_{0}(f) (\zeta) \bigr\Vert ^{p} \,d\xi\,d\eta& \leq c \vert f \vert _{\varphi,\gamma}^{p} \sum _{Q\in\mathcal{W}}\varphi \bigl( \vert Q \vert \bigr)^{p} \vert Q \vert ^{2-p}=c \vert f \vert _{\varphi,\gamma}^{p} \sum_{Q\in\mathcal {W}}h\bigl( \vert Q \vert \bigr) \\ &=c \vert f \vert _{\varphi,\gamma}^{p} s(h). \end{aligned} $$

Therefore

$$ \bigl\Vert \partial_{\bar{\zeta}}^{J}\mathcal {E}_{0}(f) \bigr\Vert _{L^{p}}\le c^{\frac{1}{p}} \vert f \vert _{\varphi,\gamma} \bigl(s(h)\bigr)^{\frac{1}{p}}. $$

The Hölder inequality then leads to

$$ \bigl\Vert T_{\Omega}\bigl[\partial_{\bar{\zeta }}^{J} \mathcal{E}_{0}(f)\bigr](\zeta) \bigr\Vert \le c \bigl\Vert \partial _{\bar{\zeta}}^{J}\mathcal{E}_{0}(f) \bigr\Vert _{L^{p}} \biggl( \int _{\Omega}\frac{d\xi\,d\eta}{ \Vert W(\zeta)-W(z) \Vert ^{q}} \biggr) ^{\frac{1}{q}}, $$

where $q= \frac{p}{p-1}$ as usual.

Using the basic property of *W*, see [2, inequality (1.14), p.12], we have

$$ \begin{aligned} \bigl\Vert T_{\Omega}\bigl[ \partial_{\bar{\zeta}}^{J}\mathcal {E}_{0}(f)\bigr](\zeta) \bigr\Vert &\le c \bigl\Vert \partial_{\bar{\zeta }}^{J} \mathcal{E}_{0}(f) \bigr\Vert _{L^{p}} \vert \gamma \vert ^{\frac{p-2}{p}}\leq c \bigl\Vert \partial_{\bar{\zeta }}^{J} \mathcal{E}_{0}(f) \bigr\Vert _{L^{p}}\psi\bigl( \vert \gamma \vert \bigr) \\ &\le c_{2} \vert f \vert _{\varphi,\gamma}\bigl(s(h) \bigr)^{\frac {1}{p}}\psi\bigl( \vert \gamma \vert \bigr) \end{aligned} $$

and

$$ \bigl\vert T_{\Omega}\bigl[\partial_{\bar{\zeta }}^{J} \mathcal{E}_{0}(f)\bigr] \bigr\vert _{\psi,\gamma}\le c_{2} \bigl\Vert \partial_{\bar{\zeta}}^{J}\mathcal{E}_{0}(f) \bigr\Vert _{L^{p}}\le c_{3} \vert f \vert _{\varphi,\gamma } \bigl(s(h)\bigr)^{\frac{1}{p}}. $$

Therefore, for every $\zeta\in\gamma$ we have

$$ \bigl\Vert H^{*}_{\gamma}f(\zeta) \bigr\Vert \le \bigl\Vert f(\zeta ) \bigr\Vert +2 \bigl\Vert T_{\Omega}\bigl[ \partial_{\bar{\zeta }}^{J}\mathcal{E}_{0}(f)\bigr](\zeta) \bigr\Vert \le \bigl\Vert f(\zeta ) \bigr\Vert +2c_{2} \vert f \vert _{\varphi,\gamma }\bigl(s(h)\bigr)^{\frac{1}{p}}\psi\bigl( \vert \gamma \vert \bigr). $$

Then

9 $$ \bigl\Vert H^{*}_{\gamma}f(\zeta) \bigr\Vert \le \Vert f \Vert _{\varphi,\gamma}+ 2c_{2} \Vert f \Vert _{\varphi ,\gamma}\bigl(s(h)\bigr)^{1/p}\psi\bigl( \vert \gamma \vert \bigr) $$

and

10 $$ \bigl\vert H^{*}_{\gamma}f \bigr\vert _{\psi,\gamma}\leq \vert f \vert _{\psi,\gamma}+2 \bigl\vert T_{\Omega}\bigl[\partial_{\bar {\zeta}}^{J}\mathcal{E}_{0}(f) \bigr] \bigr\vert _{\psi,\gamma}\leq c_{1} \frac{\varphi( \vert \gamma \vert )}{\psi( \vert \gamma \vert )} \vert f \vert _{\varphi,\gamma }+2c_{3} \vert f \vert _{\varphi,\gamma} \bigl(s(h)\bigr)^{1/p}. $$

Finally, adding (9) with (10), we obtain

$$ \bigl\Vert H^{*}_{\gamma}f \bigr\Vert _{\psi}\leq \biggl( 1+c_{1}\frac {\varphi( \vert \gamma \vert )}{\psi( \vert \gamma \vert )}+c_{2}\bigl(s(h) \bigr)^{\frac{1}{p}}\psi\bigl( \vert \gamma \vert \bigr)+c_{3} \bigl(s(h)\bigr)^{\frac{1}{p}} \biggr) \Vert f \Vert _{\varphi,\gamma} $$

or equivalently

$$ \bigl\Vert H^{*}_{\gamma}f \bigr\Vert _{\psi}\le \biggl( 1+ c_{1}\frac {\varphi( \vert \gamma \vert )}{\psi( \vert \gamma \vert )}+c_{2}\bigl(s(h) \bigr)^{\frac{1-\alpha_{\psi}}{2}}\psi\bigl( \vert \gamma \vert \bigr)+c_{3} \bigl(s(h)\bigr)^{\frac{1-\alpha_{\psi }}{2}} \biggr) \Vert f \Vert _{\varphi,\gamma}, $$

which finishes the proof. □

#### Theorem 3

*Let* *γ* *be rectifiable with length* $\mathbf{l}[\gamma]$ *and let be* *φ* *and* *ψ* *in* Φ *with* $\alpha_{\psi}<2\beta _{\varphi}-1$. *Then the Hilbert transform* $H_{\gamma}$ *is bounded from* $\mathcal {H}_{\varphi}(\gamma)$ *into* $\mathcal {H}_{\psi}(\gamma )$, *and*

11 $$ \Vert H_{\gamma} \Vert \le1+c_{1} \biggl( \frac{\varphi (\mathbf{l}[\gamma])}{\psi(\mathbf{l}[\gamma])} \biggr) +c_{2}\bigl(\mathbf {l}[\gamma] \bigr)^{\frac{1-\alpha_{\psi}}{2}}\psi\bigl(\mathbf{l}[\gamma ]\bigr)+c_{3}\bigl( \mathbf{l}[\gamma]\bigr)^{\frac{1-\alpha_{\psi}}{2}}, $$

*where* $c_{1}$, $c_{2}$, $c_{3}$ *depend only on* *φ*, *ψ*.

#### Proof

Take $p= \frac{2}{1-\alpha_{\psi}}$, then from $\alpha_{\psi}<2\beta_{\varphi}-1$ it follows that

12 $$ \frac{2}{1-\alpha_{\psi}}=p< \frac{1}{1-\beta_{\varphi}}. $$

Since *γ* is rectifiable, then it is $1+\epsilon$-summable for any $\epsilon>0$. This fact, together with (12) imply that *γ* is *h*-summable with $h(t)=\varphi(t)^{p}t^{2-p}$, which makes legitimate the use of Theorem 2. Now, the estimate (11) easily follows from (8), the simple estimate

$$ s(h)=\sum_{Q\in\mathcal{W}}\varphi\bigl( \vert Q \vert \bigr)^{p} \vert Q \vert ^{2-p}\leq c\sum _{Q\in\mathcal{W}} \vert Q \vert ^{1+p\beta_{\varphi}+1-p} $$

and Lemma 2. □

#### Remark 2

Note that the upper bound obtained in Theorem 3 generalizes and strengthens Theorem 2.6 of [6].
